# Effects of BK_Ca_ and Kir2.1 Channels on Cell Cycling Progression and Migration in Human Cardiac c-kit^+^ Progenitor Cells

**DOI:** 10.1371/journal.pone.0138581

**Published:** 2015-09-21

**Authors:** Ying-Ying Zhang, Gang Li, Hui Che, Hai-Ying Sun, Guo-Sheng Xiao, Yan Wang, Gui-Rong Li

**Affiliations:** 1 Department of Medicine, Li Ka Shing Faculty of Medicine, the University of Hong Kong, Pokfulam, Hong Kong, China; 2 Xiamen Cardiovascular Hospital, Medical School of Xiamen University, Xiamen, Fujian, 361004, China; University of Hull, UNITED KINGDOM

## Abstract

Our previous study demonstrated that a large-conductance Ca^2+^-activated K^+^ current (BK_Ca_), a voltage-gated TTX-sensitive sodium current (I_Na.TTX_), and an inward rectifier K^+^ current (I_Kir_) were heterogeneously present in most of human cardiac c-kit^+^ progenitor cells. The present study was designed to investigate the effects of these ion channels on cell cycling progression and migration of human cardiac c-kit^+^ progenitor cells with approaches of cell proliferation and mobility assays, siRNA, RT-PCR, Western blots, flow cytometry analysis, etc. It was found that inhibition of BK_Ca_ with paxilline, but not I_Na.TTX_ with tetrodotoxin, decreased both cell proliferation and migration. Inhibition of I_Kir_ with Ba^2+^ had no effect on cell proliferation, while enhanced cell mobility. Silencing KCa.1.1 reduced cell proliferation by accumulating the cells at G0/G1 phase and decreased cell mobility. Interestingly, silencing Kir2.1 increased the cell migration without affecting cell cycling progression. These results demonstrate the novel information that blockade or silence of BK_Ca_ channels, but not I_Na.TTX_ channels, decreases cell cycling progression and mobility, whereas inhibition of Kir2.1 channels increases cell mobility without affecting cell cycling progression in human cardiac c-kit^+^ progenitor cells.

## Introduction

In addition to cardiac myocytes and fibroblasts, cardiac stem cells with high growth potential, clonogenicity and pluripotency have been reported in mammalian hearts. Based on the expression of cell surface markers, cardiac stem cells have been classified into different subgroups, including side population, c-kit^+^, Sca-1^+^, Islet 1^+^, SSEA-1^+^ [1–5]. Human cardiac c-kit^+^ progenitor cells are one of the dominant members in human cardiac stem cell family. C-kit, also known as CD117 or stem cell growth factor, is the cell surface marker that has been used for stem cell isolation and enrichment from different sources [3, 6–9]. It has been reported that human cardiac c-kit^+^ progenitor cells have the capability to differentiate into three cardiac lineages, i.e. cardiomyocytes, smooth muscle and endothelial cells [10–12]. The *in situ* stimulation of c-kit^+^ progenitor cell growth or injection of expanded c-kit^+^ progenitor cells to the infarct area has been reported to improve cardiac repair, heart function and survival after myocardial infarction [13, 14].

It is well recognized that ion channels play a crucial role in controlling electrophysiology and excitation-contraction coupling in cardiomyocytes in the heart. Our recent study has demonstrated that ion channels regulate cell cycling progression in human cardiac fibroblasts [15]. Although we demonstrated that a large conductance Ca^2+^-activated K^+^ current (BK_Ca_), an inwardly-rectifying K^+^ current (I_Kir_), and a voltage-gated tetrodotoxin-sensitive Na^+^ currents (I_Na.TTX_), were heterogeneously expressed in most (61–86%) of human cardiac c-kit^+^ progenitor cells [16], the potential physiological roles of these channels are not understood. The present study was to investigate the roles of these functional ion channels in regulating cell cycling progression and mobility in human cardiac c-kit^+^ progenitor cells with the approaches including cell proliferation and migration assays, flow cytometry, siRNA, RT-PCR, and Western blot analysis.

## Materials and Methods

### Cell culture

Human cardiac c-kit^+^ cells were isolated from atrial specimens obtained from coronary artery bypass surgery with the modified procedure as described previously [3, 11, 16], and the procedure of tissue collection was approved by the Ethics Committee of the University of Hong Kong (UW-10-174, S1 File), with written consent from patients as described previously [16]. In the previous report, we demonstrated that human cardiac c-kit^+^ cells expressing the stem cell markers CD29 and CD105 were >99%, in which the hematopoietic stem cell markers CD34 and CD45, and adult somatic cell marker CD8A were present in a very limited population (<10%), and hematopoietic stem cell markers CD34 and CD45 were mostly absent [16], consistent with the previous reports by other research groups [3, 11]. The cells were cultured in Iscove’s Modified Dulbecco’s Medium (IMDM) containing 10% FBS, 100 U/ml penicillin, 100 μg/ml streptomycin, 2 mM L-glutamine, 0.1 mM 2-mercaptoethanol, 5 ng/ml human basic fibroblast growth factor, 5 ng/ml human epidermal growth factor [16].

### Chemicals and reagents

Mouse monoclonal anti-KCa1.1 and anti-Kir2.1 antibodies were from UC Davis (www.neuromab.org). Goat anti-mouse IgG horseradish peroxidase (HRP) and mouse monoclonal anti-GAPDH antibodies were from Santa-Cruz Biotechnology Inc. (Santa Cruz, CA http://www.scbt.com). Epithelial growth factor (EGF), basic fibroblast growth factor (bFGF), propidium iodide (PI), lipofectamine 2000, Triton X-100 and Tween 20 were purchased from Invitrogen (Invitrogen, Hong Kong, China). [^3^H]-thymidine was from GE Healthcare Life Sciences (Hong Kong, China). Other reagents were obtained from Sigma-Aldrich (St. Louis, MO, USA).

### Whole-cell patch recording

Human cardiac c-kit^+^ progenitor cells (passages 2–4) were trypsinized when cell grew to 70–80% confluence used for ionic current recordings with a whole-cell patch voltage-clamp technique (at room temperature, 23–25°C) using an EPC-9 amplifier and Pulse software (Heka, Lambrecht, Germany) as described previously [16].

### Cell proliferation assays

Cell proliferation was determined by 3-(4,5-Dimethyl-thiazol-2-yl)-2,5-diphenyl tetrazolium bromide (MTT) assay and DNA incorporation with [^3^H]-thymidine to evaluate the effects of ion channel blockade or ion channel silence on cell proliferation with procedure described previously [17, 18]. For MTT assay, cells were plated into 96-well plates at a density of ~4000 cells per well (2×10^4^ cells/ml) in 200 μl complete culture medium. After 8 h culture, ion channel blockers or vehicle (control) were applied for additional 48 h incubation, and PBS-buffered MTT (5 mg/ml) solution (20 μl) was added for additional 4 h incubation. The mixture of culture medium with MTT was then removed, and DMSO (100 μl) was added to each well to dissolve the formazan crystals formed in cells attached at the bottom. The plates were protected from light with agitation for 30 min at room temperature. The absorbance was read at wavelength 570 nm with a reference filter of 630 nm using a Quant microplate spectrophotometer (Bio-Tek Instruments) for quantitative evaluation. In experiments with siRNA molecules targeting to different ion channels, cells were plated into 96-well plates at a density of ~4000 cells per well. The siRNA molecules were transfected into the cells for 8 h, and the cells were cultured for additional 64 h. MTT solution (20 μl per well) was then added. Results were standardized using control group values.

[^3^H]-thymidine incorporation assay was performed using 96-well plates with seeding ~4000 cells per well in 200 μl complete culture medium. The cells were cultured for 8 h, and then the culture medium was changed to that containing ion channel blockers, or siRNA molecules. After 48 h incubation with ion channel blockers or 72 h after siRNA transfection, [^3^H]-thymidine was added into each well at the concentration of 1 μCi (0.037 MBq). [^3^H]-thymidine (1 μCi) was added into each well with an additional 12 h incubation, and the cells were harvested and transferred to a nitrocellulose-coated 96-well plate via suction. Nitrocellulose membrane was washed with H_2_O, and the plate was air dried at 50°C overnight. Liquid scintilla (20 μl/well) was then added to each well. Counts per min (CPM) were read by a TopCount microplate scintillation and luminescence counter (PerkinElmer, Waltharn, MA).

### Cell mobility determination

Cell migration was determined using a wound healing method and chemotaxis assay with a transwell system to investigate the potential effect of ion channels on cell motivation in human cardiac c-kit^+^ progenitor cells with procedure described previously [19, 20]. The wound healing assay was conducted when the cells grew to total confluence in 6-well plates. A standard wound was created by scratching the cell monolayer with a sterile 200 μl plastic pipette tip. Line makers were made at the bottom of plates to indicate the wound edges. After removing cell fragments by washing cell monolayer gently with PBS, the cells were incubated at 37°C with the medium containing 1% FBS and ion channel blockers (not for the cells transfected with siRNA molecules) for 8 h. Then the defined areas of the wound gap were photographed under a phase contrast microscope (Olympus, Tokyo, Japan). The migrated cells on the images were counted to assess cell mobility under different conditions of treatments.

Transwell assay with a modified Boyden chamber with 8 μm-pore polycarbonate membranes (Corning Inc., Corning, NY, USA) was made to determine cell migration following the procedure described previously [19] to exclude the potential contamination of cell migration by proliferated cells. The chambers were pre-coated with 600 μl serum-free medium for at least 1 h. After the pre-coated medium was removed, ~5000 viable human cardiac c-kit^+^ cells were plated into the upper chamber in 200 μl medium containing 1% FBS with or without ion channel blockers, and the lower chamber was added 600 μl medium with 1% FBS. The plates were incubated at 37°C in 5% CO_2_ for 8 h. Then the chambers were washed with PBS for three times, fixed with formaldehyde for 15 min at room temperature, and stained with crystal violet for 15 min. After washing with PBS to thoroughly remove the dye, non-migrated cells on the upper surface of the membrane were scraped off by cotton swabs. The migrated cells on the lower surface of the membrane were counted under a microscope.

### Cell cycling progression analysis

Flow cytometry (FC500, Beckman Coulter) was used to determine cell cycling progression in human cardiac c-kit^+^ progenitor cells with procedure as described previously [17, 18]. Briefly, the cells were plated in 100 mm cell culture dishes at a density of 6×10^3^ cells/cm^2^, cultured for 8 h in complete culture medium, and synchronized to G0/G1 phase with a cultured medium containing 1% FBS for 12 h. The cells were then cultured in normal culture medium with treatment of ion channel blockers for 60 h or siRNA transfection for 72 h. The cells were lifted using 0.125% trypsin, washed with PBS, and fixed with ice-cold ethanol (75%) at −20°C (72 h). Then ethanol was removed by centrifugation, and cell pellets were washed with PBS twice. Cells were incubated in a propidium iodide/PBS staining buffer (20 μg/ml propidium iodide, 100 μg/ml RNase A, and 0.1% Triton X-100) at 37°C for 30 min. Data were acquired using CellQuest software, and the percentages of G0/G1, S, and G2/M phase cells were calculated with MODFIT LT software.

### Small interference RNA

Small interference RNA (siRNA) technique was adopted to silence the related ion channels with the procedure described previously [17, 18]. Briefly, siRNA molecules targeting human KCa1.1 (sc-42511) and Kir2.1 (sc-42612) were purchased from Santa Cruz Biotechnology. These siRNA molecules are pools of 3 target-specific 20–25 nucleotides designed to silence corresponding gene expression. Lipofectamine 2000 reagent (Invitrogen) was used for siRNA transfection. Total RNA and protein were extracted and evaluated by RT-PCR and Western-blot respectively after 72 h transfection. Membrane potential and currents were recorded in current clamp mode and voltage-clamp mode, respectively. Proliferation and migration assays, and flow cytometry analysis were conducted after 72 h siRNA transfection.

### Reverse transcription and polymerase chain reaction

Total RNA of human cardiac c-kit^+^ positive progenitor cells was isolated using the TRIzol method (Invitrogen). Reverse transcription (RT) was performed with the RT system (Promega Corp., Madison, WI, USA) protocol in a 20-μ1 reaction mixture with the procedure as described previously [17, 18]. After the RT, the reaction product (cDNA) was used for polymerase chain reaction (PCR). The cDNA was kept at −80°C for long-time storage.

Primers used in the present study were adopted from our previous report [16]. PCR was performed with the Promega PCR Core System I using a DNA thermal cycler (Mycycler; Bio-Rad Laboratories, Hercules, CA) as described previously [16]. The PCR products, amplified cDNA bands, were analyzed by 1.3% agarose gel electrophoresis, and visualized in ethidium bromide-stained gel illuminated with UV light. Quantitative evaluation and imagination was conducted via the Chemi-Genius Bio Imaging System (Syngene, Cambridge, UK).

### Western blotting analysis

Western blot analysis was performed to determine protein expression of with the procedure as described previously [18]. Briefly, cells lysates were extracted via a modified RIPA buffer, and cell lysates (50 μg) were mixed with sample buffer and denatured by heating to 70°C for 10 min. Samples were resolved via SDS-PAGE and transferred to nitrocellulose membranes. Membranes were blocked with 5% nonfat milk in Tris-buffered saline with Tween (TTBS) and then probed with primary antibody (mouse monoclonal anti-KCa1.1, anti-Kir2.1, or anti-GAPDH) at 4°C overnight with agitation. After washing with TTBS, the membranes were incubated with goat anti-mouse IgG-horseradish peroxidase (HRP) at 1:4,000 dilution in TTBS at room temperature for 1 h. Membranes were washed again with TTBS and then processed to develop X-ray film using an enhanced chemiluminescence detection system (GE Healthcare). The expression of GAPDH levels was used as an internal control to standardize the relative levels of target protein. The relative band intensities of Western blot were measured by quantitative scanning densitometer and image analysis software (Bio-1D version 97.04).

### Statistical analysis

Results were expressed as mean ± SEM. Unpaired Student’s t-test was used as appropriate to evaluate the statistical significance of differences between two group means, and analysis of variance was used for multiple groups. A value of P<0.05 was considered statistically significant.

## Results

### Inhibition of membrane currents by specific blockers in human cardiac c-kit^+^ progenitor cells

**Fig 1:**
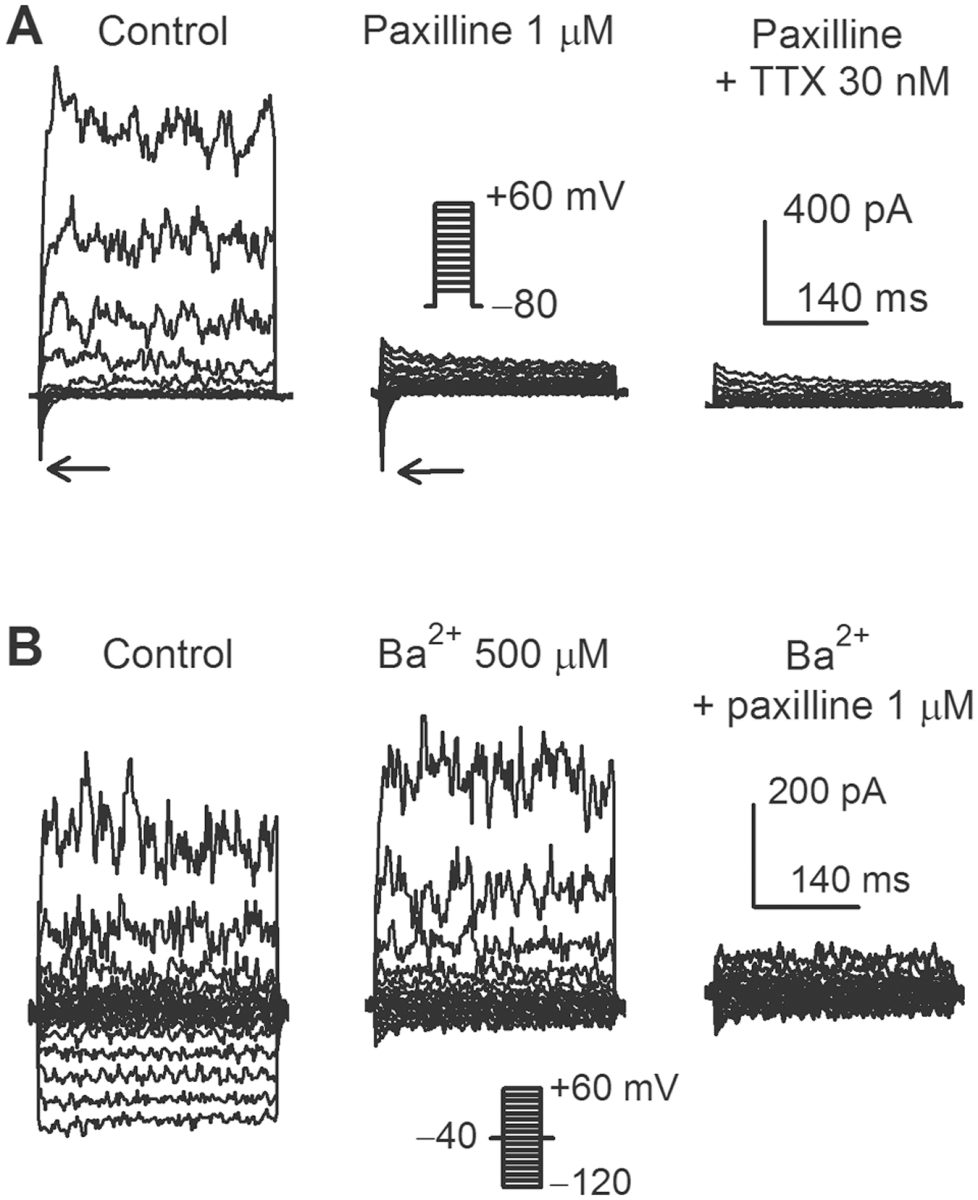
Inhibition of membrane current by ion channel blockers in human cardiac c-kit^+^ progenitor cells. **(A)**: BK_Ca_ and I_Na.TTX_ were co-expressed in a human cardiac c-kit^+^ progenitor cell, and inhibited respectively by 1 μM paxilline and 30 nM TTX. **(B)**: I_Kir_ and BK_Ca_ were co-expressed in a typical human cardiac c-kit^+^ progenitor cell, and inhibited respectively by 500 μM Ba^2+^ and 1 μM paxilline.

Our previous study demonstrated that BK_Ca_ (encoded by KCa1.1), I_Na.TTX_ (encoded by Nav1.3 and Nav1.6) and I_Kir_ (encoded by Kir2.1) were heterogeneously co-expressed in human cardiac c-kit^+^ progenitor cells [16], which are inhibited by corresponding blockers as shown in Fig 1. BK_Ca_ and I_Na.TTX_ were co-expressed in a human cardiac c-kit^+^ progenitor cell and inhibited respectively by the BK_Ca_ inhibitor paxilline (1 μM) and tetrodotoxin (TTX, 30 nM) (Fig 1A), while I_Kir_ and BK_Ca_ were co-expressed in another representative cell and suppressed respectively by Ba^2+^ (500 μM) and paxilline (Fig 1B). Similar results were obtained in other 10 cells for each treatment.

### Effects of ion channel blockers on cell proliferation

**Fig 2:**
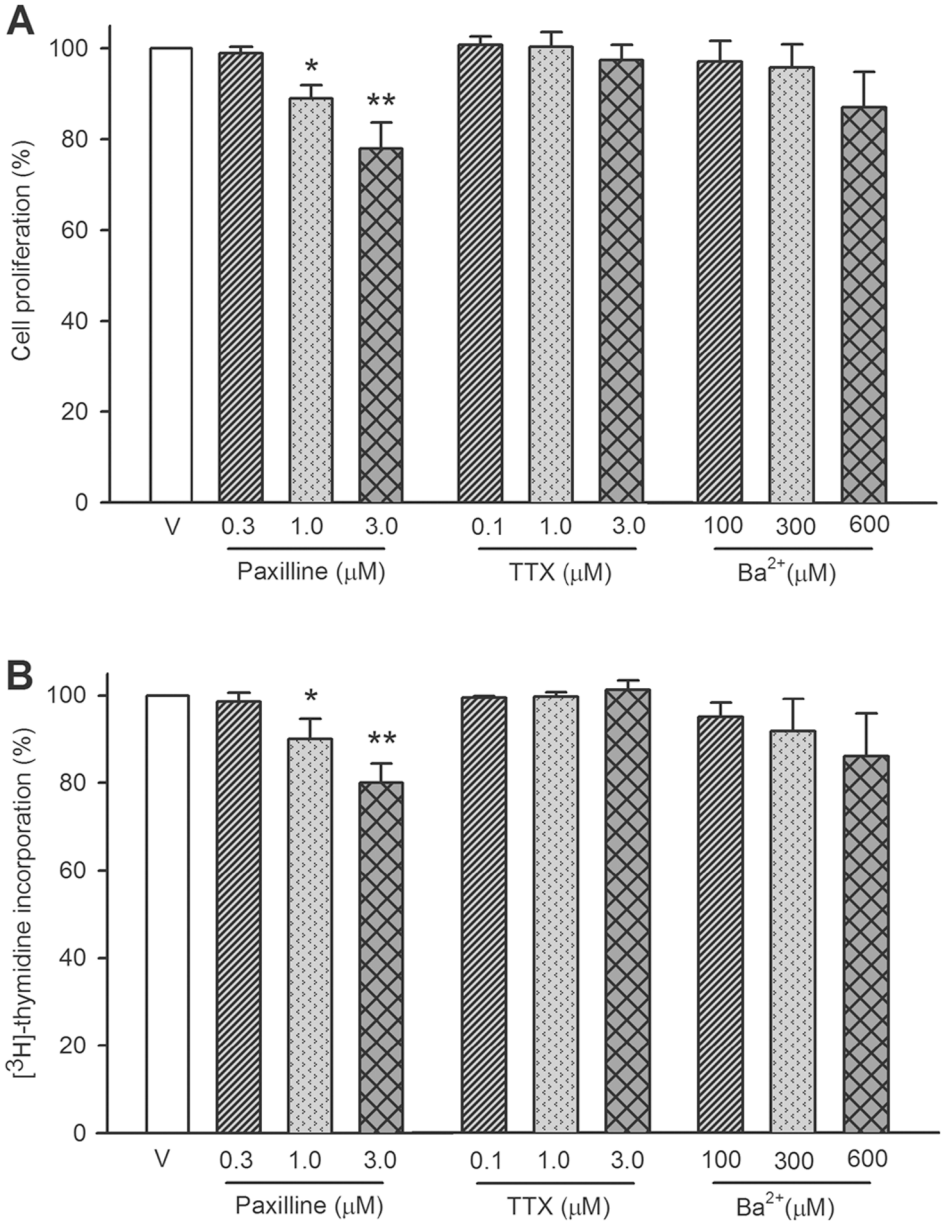
Effects of ion channel blockers on cell proliferation in human cardiac c-kit^+^ progenitor cells. **(A)**: cell proliferation was assessed by MTT assay in cells treated with vehicle (V), paxilline, TTX or Ba^2+^ at concentrations as indicated (n = 8, *P<0.05, **P<0.01 vs. vehicle control). **(B)**: [^3^H]-thymidine incorporation assay was conducted in cells treated with paxilline, TTX or Ba^2+^ at different concentrations (n = 6, *P<0.05, **P<0.01 vs. vehicle control).

To determine whether blockade of ion channels would affect cell proliferation, MTT assay was initially used in human cardiac c-kit^+^ progenitor cells. The cells were treated with paxilline (0.1–3 M) to block BK_Ca_, TTX (0.1–3 M) to block I_Na.TTX_, and Ba^2+^ (100–600 M) to block I_Kir_ for 48 h. Fig 2A shows the percentage values of cells in the absence or presence of different ion channel blockers. The cell proliferation was inhibited by 1 and 3 M paxilline (n = 8, P<0.05 or P<0.01 vs. control), but not by TTX or Ba^2+^ (n = 8, P = NS).

Similar results were obtained with [^3^H]-thymidine incorporation assay (Fig 2B). Paxilline (1 and 3 M), but not TTX and Ba^2+^, significantly reduced DNA synthesis rate in human cardiac c-kit^+^ progenitor cells (n = 6, P<0.05 or 0.01 vs. control). These results suggest that the inhibition of BK_Ca_ decreases the proliferation of human cardiac c-kit^+^ progenitor cells, while blockade of I_Na.TTX_ or I_Kir_ had no significant effect on cell proliferation.

### Blockade of ion channels on cell migration

**Fig 3:**
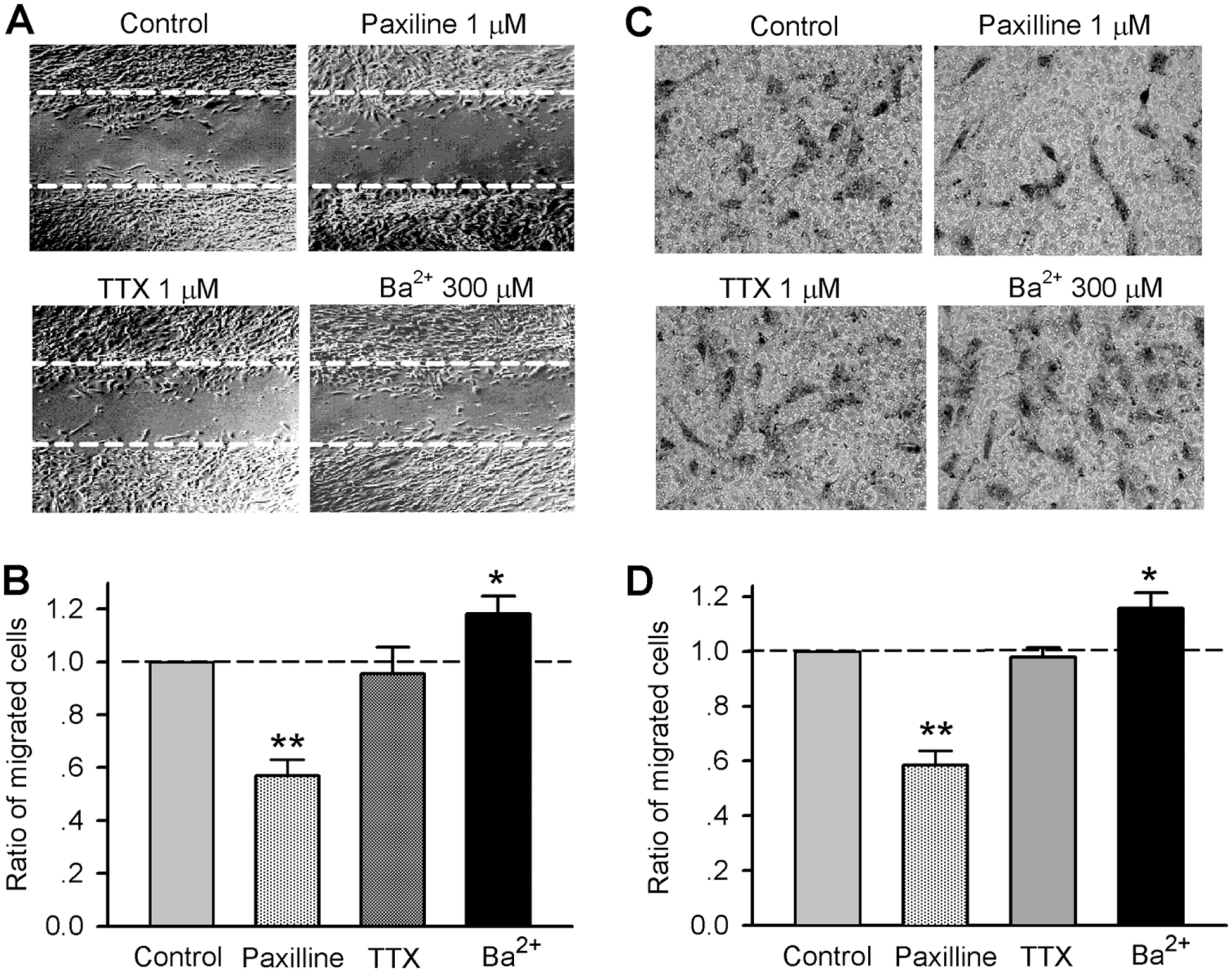
Effects of ion channel blockers on cell migration in human cardiac c-kit^+^ progenitor cells. **(A)**: Cell images with acellular wound. The broken lines indicate the initial wound produced with a pipette tip in cells treated with vehicle (control), paxilline (1 M), TTX (1 M), or Ba^2+^ (300 μM). **(B)**: Ratio of migrated cells in human cardiac c-kit^+^ progenitor cells treated with vehicle, 1 μM paxilline, 1 μM TTX or 300 μM Ba^2+^ (n = 6; *P<0.05, **P<0.01 vs. vehicle control). **(C)**: Images of migrated human cardiac c-kit^+^ progenitor cells on lower surface of the transwell membrane in cells treated with vehicle (control), paxilline (1 M), TTX (1 M) or 300 μM Ba^2+^. **(D)**: Ratio of migrated human cardiac c-kit^+^ progenitor cells on the lower membrane in cells treated with vehicle (control), paxilline (1 M), TTX (1 M) or 300 μM Ba^2+^ (n = 5; *P<0.05, **P<0.01 vs. vehicle control).

To examine whether ion channels would regulate cell migration in human cardiac c-kit^+^ progenitor cells, wound healing and chemotaxis assays were conducted in cells treated with different ion channel blockers. Fig 3A shows the wound healing images in cells treated with paxilline (1 μM), TTX (1 μM), or Ba^2+^ (300 μM) for 8 h. Fig 3B illustrates the ratio of migrated cells into the acellular area in different treatments. Blockade of BK_Ca_ channels with paxilline, but not I_Na.TTX_ with TTX, significantly inhibited cell migration (n = 7, P<0.01 vs. vehicle control). Interestingly, blockade of I_Kir_ with Ba^2+^ increased cell migration (n = 6, P<0.05 vs. control).

Fig 3C displays the chemotaxis assay with a HTS Transwell system. The cells migrated to the lower surface of the membrane were reduced with treatment of 1 μM paxilline, whereas increased with treatment of 300 μM Ba^2+^. TTX treatment did not affect cell mobility. Fig 3D illustrates the ratio of migrated cells on lower surface of the membrane. The ratio of migrated cells was decreased by 1 M paxilline (n = 5, P<0.01 vs. control), while increased by 300 μM Ba^2+^ (n = 5, P<0.05 vs. control). TTX (1 μM) had no effect on cell migration. These results suggest that blockade of BK_Ca_, but not I_Na.TTX_, decreases cell migration, whereas inhibition of I_Kir_ increases cell migration in human cardiac c-kit^+^ progenitor cells.

### Silence of ion channels with corresponding siRNA molecules

To exclude the potential nonspecific effects of BK_Ca_ and I_Kir_ blockers on cell proliferation and/or migration, siRNA molecules targeting KCa1.1 gene (for BK_Ca_), and Kir2.1 gene (for I_Kir_) were employed in human cardiac c-kit^+^ progenitor cells. The experiment of silencing I_Na.TTX_ was not performed, because no effect was observed on cell proliferation or migration in cells treated with0.1–3 μM TTX, and the concentration was much higher than that for blocking the current.

**Fig 4:**
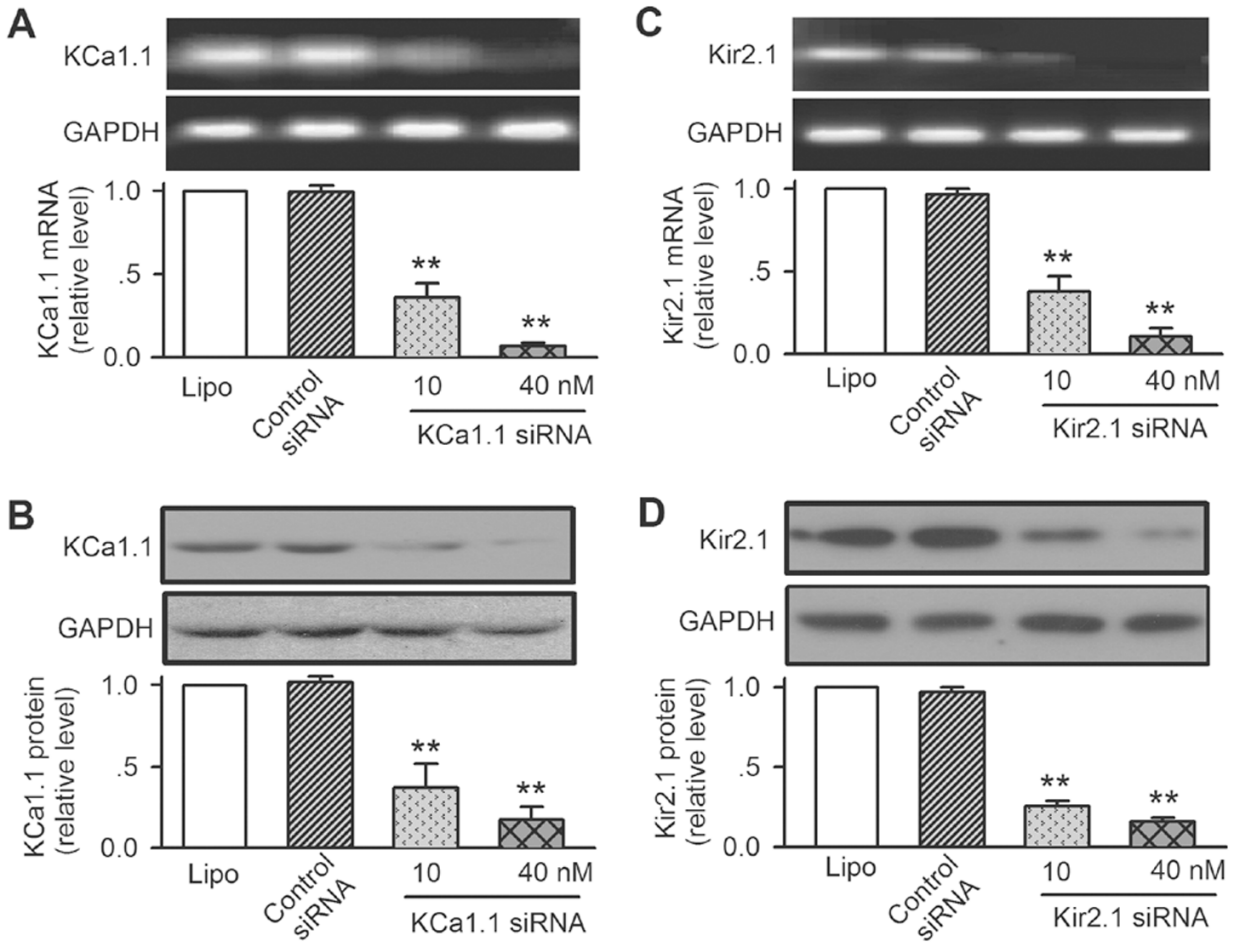
Effects of specific siRNA molecules on expression of ion channel genes and proteins in human cardiac c-kit^+^ progenitor cells. **(A)**: PCR images and relative level of KCa1.1 mRNA in cells treated with lipofectamine 2000 (Lipo), control siRNA, or KCa1.1 siRNA. **(B)**: Western blots and relative level of KCa1.1 protein in cell treated with lipofectamine 2000, control siRNA, or KCa1.1 siRNA. **(C)**: PCR images and relative level of Kir2.1 mRNA in cells treated with lipofectamine 2000, control siRNA, or Kir2.1 siRNA. **(D)**: Western blots and relative level of Kir2.1 protein in cell treated with lipofectamine 2000, control siRNA, or Kir2.1 siRNA (n = 6 for each group, **P<0.01 vs. control siRNA).

Fig 4A and 4C illustrate the RT-PCR images and the relative mRNA levels of KCa1.1 and Kir2.1 in cells transfected with the corresponding siRNA molecules (10 or 40 nM) for 72 h. KCa1.1 siRNA and Kir2.1 siRNA molecules significantly reduced the corresponding mRNA levels. The mRNA was reduced to 7.0 ± 1.8% of control for KCa1.1 and 10.5 ± 5.4% for Kir2.1 in cells transfected with corresponding siRNA molecules (n = 5, P<0.01 vs. control siRNA 40 nM).

Western blots of KCa1.1 and Kir2.1 channels were determined in cells transfected with the corresponding siRNA molecules. Significant reduction of protein level was observed in cells transfected with siRNA molecules targeting to KCa1.1 (Fig 4B) or Kir2.1 (Fig 4C). The mean relative protein level was reduced to 17.3 ± 8.2% of control for KCa1.1, and 16.0 ± 2.5% for Kir2.1 in cells transfected with 40 nM corresponding siRNA molecules (n = 6, P<0.01 vs. control siRNA 40 nM).

Membrane potential, BK_Ca_ and I_Kir_ currents were recorded in human cardiac c-kit^+^ progenitor cells transfected with siRNA molecules. The membrane potential recorded in current clamp mode. BK_Ca_ current recorded with the voltage protocol as shown in Fig 1A, and I_Kir_ was recorded with the voltage protocol as shown in Fig 1B. In cells transfected with 40 nM control siRNA, the membrane potential was -51.3 ± 2.5 mV (n = 25, with adjustment of 15 mV liquid junctional potential in pipette solution). BK_Ca_ at +60 mV was 4.1 ± 1.2 pA/pF (n = 18), and I_Kir_ at -100 mV was -2.8 ± 0.5 pA/pF (n = 17).

In cells transfected with 40 nM KCa1.1 siRNA, BK_Ca_ current at +60 mV was reduced to 1.3 ± 0.7 pA/pF (n = 14, P<0.01 vs. control siRNA) and the membrane potential was slightly depolarized (-47.5 ± 3.7 mV, n = 15, P = NS vs. control siRNA). Nonetheless, in cells transfected with 40 nM Kir2.1 siRNA, I_Kir_ at -100 mV was decreased (to -0.8 ± 0.7 pA/pF, n = 13, P<0.05 vs. control siRNA) and the membrane potential was significantly depolarized (to -23.9 ± 2.5 mV, n = 16, P<0.01 vs. control siRNA). These results indicate that silencing KCa1.1 channels reduces BK_Ca_ current with slight membrane depolarization, whereas silencing Kir2.1 channels decreases I_Kir_ with significant membrane depolarization.

### Effects of silencing KCa1.1 and Kir2.1 on cell proliferation and cell cycling progression

**Fig 5:**
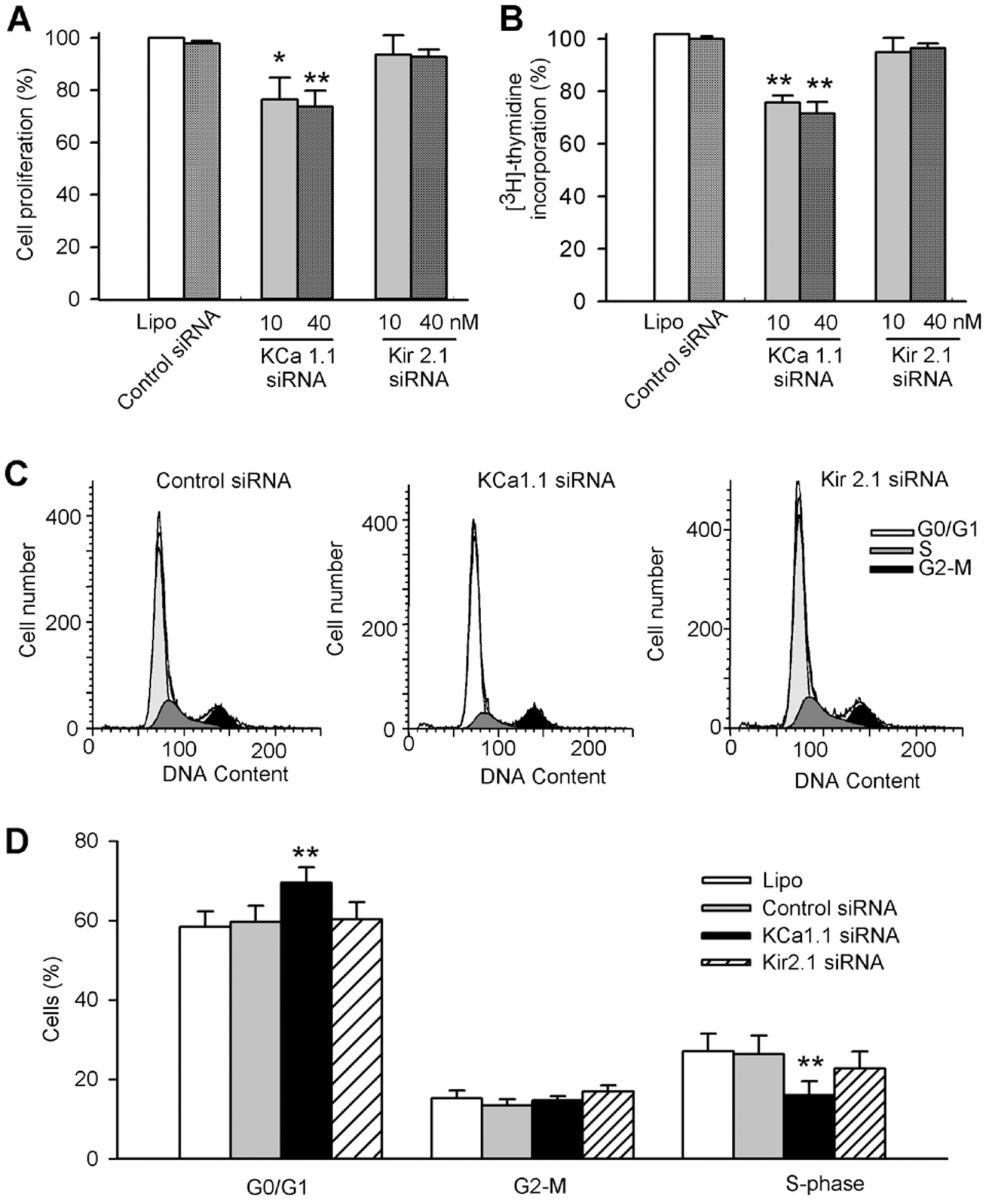
Effects of silencing KCa1.1 or Kir2.1 on cell proliferation in human cardiac c-kit^+^ progenitor cells. **(A)**: Cell proliferation was assessed by MTT assay in cells treated with lipofectamine 2000 (Lipo) or transfected with control siRNA (40 nM), KCa1.1 siRNA or Kir2.1 siRNA (10 nM and 40 nM). **(B)**: [^3^H]-thymidine incorporation assay was conducted in cells treated with lipofectamine 2000 or transfected with control siRNA, KCa1.1 siRNA, or Kir2.1 siRNA (n = 6 for each group, *P<0.05, **P<0.01 vs. control siRNA). (C). Flow cytometry graphs in cells transfected with control siRNA, KCa1.1 siRNA, or Kir2.1 siRNA. **(D)**: Mean values of different cycle phases in cells treated with lipofectamine, control siRNA, KCa1.1 siRNA, or Kir2.1 siRNA (40 nM each group, n = 6,*P<0.05, **P<0.01 vs. control siRNA).

The effects of silencing KCa1.1 or Kir2.1 channels on cell proliferation were determined with MTT and [^3^H]-thymidine incorporation assays in human cardiac c-kit^+^ progenitor cells transfected with the corresponding siRNA molecules. Cell proliferation (Fig 5A) was significantly reduced in cells transfected with KCa1.1 siRNA (n = 6, P<0.05 or P<0.01 vs. control), but not with Kir2.1 siRNA (n = 4, P = NS). Similar results were obtained with [^3^H]-thymidine incorporation assay (Fig 5B). DNA synthesis rates were significantly reduced by KCa1.1 siRNA, not by Kir2.1 siRNA. These results confirm that BK_Ca_, but not I_Kir_, participates in the regulation of cell proliferation.

The effects of silencing KCa1.1 and Kir2.1 channels on cell cycling progression were determined with flow cytometry analysis (Fig 5C) in human cardiac c-kit^+^ progenitor cells transfected with corresponding siRNA molecules. Fig 5D illustrates the mean percentage values of cycling phases in cells transfected with 40 nM siRNA molecules targeting to KCa1.1 or Kir2.1. Portion of G0/G1 population was increased from 59.7 ± 4.0% of cells transfected with control siRNA to 69.5 ± 3.9% with KCa1.1 siRNA (n = 8, P<0.01 vs. control siRNA), while Kir2.1 siRNA molecules had no effect on cell cycling progression. These results indicate that BK_Ca_, but not I_Kir_, regulates cell cycling progression by accumulating cells at G0/G1 phase in human cardiac c-kit^+^ progenitor cells.

### Effects of silencing KCa1.1 and Kir2.1 channels on cell migration

**Fig 6:**
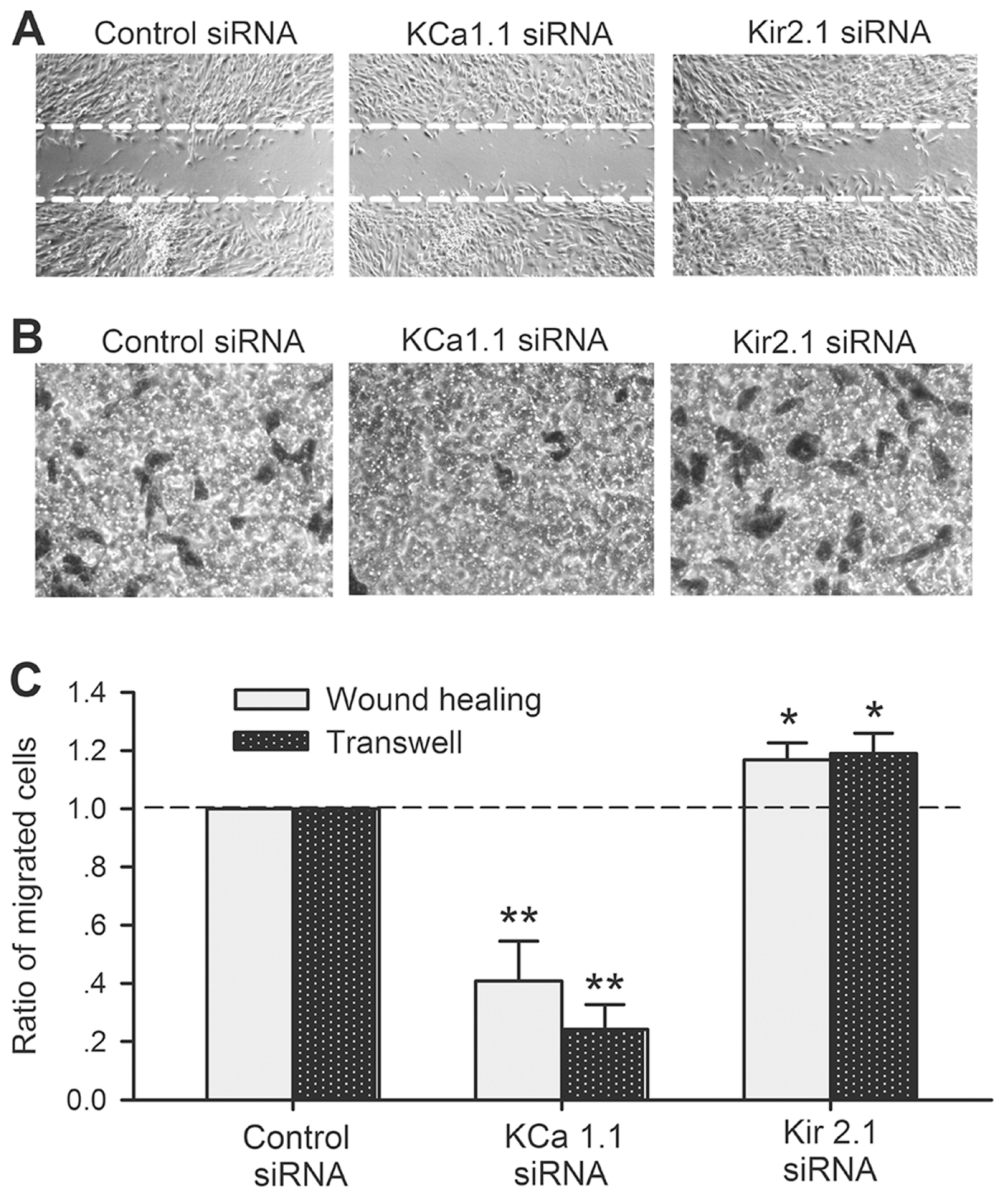
Effects of silencing KCa1.1 or Kir2.1 on cell migration in human cardiac c-kit^+^ progenitor cells. **(A)**: Images of human cardiac c-kit^+^ progenitor cells with wound-healing migration assay in confluent cells transfected with control siRNA, KCa1.1 siRNA or Kir2.1 siRNA (40 nM for each group). **(B)**: Images of migrated human cardiac c-kit^+^ progenitor cells to the lower membrane in cells transfected with control siRNA, KCa1.1 siRNA or Kir2.1 siRNA (40 nM for each group). **(C)**: Mean values of ratio of migrated cells in cells transfected with control siRNA, KCa1.1 siRNA or Kir2.1 siRNA (40 nM for each group, n = 5 for each group, *P<0.05, **P<0.01 vs. control siRNA).

The effects of BK_Ca_ and I_Kir_ on cell migration were confirmed with wound healing assay Fig 6A) and transwell assay (Fig 6B) in cells transfected with 40 nM siRNA molecules targeting to KCa1.1 or Kir2.1. The mean values of the ratio of cells migrated to the acellular area or the lower membrane surface of transwell were reduced in human cardiac c-kit^+^ progenitor cells transfected with KCa1.1 siRNA (n = 5, P<0.01 vs. control siRNA), while increased in cells transfected with Kir2.1 siRNA molecules (n = 5, P<0.05 vs. control siRNA). These results indicate that silencing BK_Ca_ (KCa1.1) inhibits cell mobility, while silencing I_Kir_ (Kir2.1) increases cell mobility in human cardiac c-kit^+^ progenitor cells.

## Discussion

It is generally recognized that ion channels play important roles in maintaining physiological homeostasis. In excitable cells, ion channels initiate action potentials and conduct the excitation impulse in excitable cells (e.g. neuronal cells, muscle cells, etc.) to generate the excitation-contraction coupling in muscle cells, and the excitation-secretion coupling in gland cells. However, in proliferative cells, ion channels are considered to participate in regulating cell proliferation and mobility in different types of cells [17, 21].

Early in 1984, DeCoursey and colleagues first reported the regulation of cell growth by ion channels in human T lymphocytes [22]. Afterwards, the roles of specific ion channels in modulating cell proliferation are gradually established. Blockade of Kv and/or K_Ca_ channels is demonstrated to inhibit proliferation in glial cells, lymphocytes, endothelium, breast and prostate cancer cells [23], and in bone marrow-derived MSCs from mouse [18], rat [17] and human [24], mouse cardiac c-kit^+^ progenitor cells [25], rat vascular smooth muscle cells [20, 26], and also in rat and human cardiac fibroblasts [15, 27]. Moreover, ion channels are found to regulate cell motility [28].

Our previous study reported that BK_Ca_, I_Kir_, and I_Na.TTX_, are heterogeneously expressed in most (61–86%) of human cardiac c-kit^+^ cells [16]. In the present study, we demonstrated the new information that blocking or silencing BK_Ca_ channels inhibited both cell proliferation and migration, while inhibiting or silencing Kir2.1 channels increased cell migration without affecting cell proliferation. However, blockade of I_Na.TTX_ had no effect on either cell proliferation or migration in human cardiac c-kit^+^ progenitor cells.

Although BK_Ca_ channels have been demonstrated to participate in the regulation of cell proliferation in several types of cells, including human cardiac fibroblast [15], human preadipocytes [29], endothelial cells [30], and human cardiac c-kit^+^ progenitor cells observed in the present study, inhibition of BK_Ca_ is found to have little effect on cell proliferation in human bronchial smooth muscle cells [31], or MCF-7 cells [32], indicating that regulation of cell proliferation by BK_Ca_ channels is cell-type dependent.

The Ca^2+^-activated K^+^ (K_Ca_) channels, including BK_Ca_ (KCa1.1), SK_Ca_ (e.g. KCa2.3), and IK_Ca_ (KCa3.1) channels are reported to regulate cell mobility. Inhibition of K_Ca_ channels usually reduces cell migration; however, the epithelial restitution is accelerated when KCa3.1 channel is inhibited in intestinal epithelial cells [33]. KCa1.1 channels are only required for migration in gloma cells, but not in microglia cells [34, 35]. In the present study, we demonstrated that blockade of BK_Ca_ with paxilline or silencing BK_Ca_ with specific siRNA molecules inhibited cell migration in human cardiac c-kit^+^ progenitor cells. The reports from ours and others suggest that the contribution of different K_Ca_ channels to cell migration is also cell-type specific. The modulation of cell migration by ion channels is believed to be related to the regulation of cell membrane potential and cell volume and/or the nonconductive properties of ion channels.

It is well recognized that the Kir2 inward-rectifier K^+^ channel family including Kir2.1 is expressed in both excitable and non-excitable cells and the primary function of Kir2 maintains a hyperpolarized membrane potential. Cardiac I_K1_ (mainly encoded by Kir2.1) has been well studied in human cardiac myocytes [36–39]. Dysfunction of I_K1_/Kir2.1 channels depolarized the resting membrane potential, caused a delayed repolarization of action potential, thus induced serious cardiac arrhythmia [40, 41]. Patients with Andersen Syndrome are characterized with Kir2.1 mutation [42]. The effects of Kir2.1 on cellular functions in non-excitable cells are somewhat controversial. Kir2.1 was reported to be necessary for differentiation of myoblasts [43] and play a role in the fusion of mono-nucleated myoblasts to form a multinucleated skeletal muscle fiber [44]. In human endothelial progenitor cells, inhibition of Kir2.1 was found to enhance cell proliferation [45]. A recent report demonstrated that blocking Kir2.1 increased proliferation, and decreased the migration induced by IL-4, IL-10 or ATP in cultured rat microglial cells [46]. Interestingly, we demonstrated the blockade of I_Kir_ with Ba^2+^ or silencing Kir2.1 channels with siRNA depolarized the membrane potential, and stimulated cell migration without affecting proliferation in human cardiac c-kit^+^ progenitor cells. The effect is similar to the inhibition of KCa3.1 channels in intestinal epithelial cells [33]. The results from ours and others support the notion that the Kir2.1 regulation of non-excitable cell functions depends on cell types.

I_Na_ plays an important role in determining rapid upstroke of cardiac action potential. TTX-insensitive Na_v_1.5 channels are predominantly present in the heart and code for I_Na_ in cardiomyocytes, while TTX-sensitive Na_V_1.2, Na_V_1.3, Na_V_1.6, or Na_V_1.7 channels are mainly reported in neuronal cells and code for I_Na_ in brain. We found that I_Na.TTX_ (encoded by Nav1.3 and Nav1.6) was also present in human cardiac c-kit^+^ progenitor cells [16]. Although the previous studies reported that blockade of TTX-insensitive voltage-gated sodium channels (I_Na_, encoded by Nav1.5) was found to reduce proliferation or migration in gastrointestinal epithelial cells [47, 48], we did not find any effect of inhibiting I_Na.TTX_ (with concentrations much higher than that for inhibiting the current) on cell proliferation or migration in human cardiac c-kit^+^ progenitor cells in the present study. These results suggest that TTX-sensitive I_Na_ (e.g. Na_V_1.2, Na_V_1.3, Na_V_1.6, or Na_V_1.7 channels), unlike the TTX-insensitive I_Na_ (e.g. Nav1.5), may not have effect on cell cycling progression and/or mobility.

It is believed that cell proliferation and mobility are strictly regulated by multiple mechanisms. Thus, ion channel-mediated regulation of cell cycling progression may not be the sole determinant. During cell proliferation, an increase of cell volume is required, which needs the active participation of ion transport through appropriate ion channels across the cell membrane [21, 49]. Though the detailed mechanisms underlying cell growth regulation by ion channels remain to be further studied, ion channels are generally believed to modulate cell proliferation by regulating cell volume, membrane potential and/or driving force for Ca^2+^, and also protein-protein interaction [50]. A number of studies demonstrate that membrane potential changes during cell cycling progression [17, 46, 51, 52]; however, in the present study, proliferation was not affected in cells with depolarized membrane potential by silencing Kir2.1 channels, in which, however, cell mobility was increased.

The ability of homing to areas of acute or chronic myocardial injury is very important for human cardiac c-kit^+^ progenitor cells in the treatment of injury therapy. Recent studies have reported that ion channels are closely involved in the regulation of cell migration in many types of cells, including human mesenchymal stem cells [53], monocytes [54], colon cancer cells [55], pancreatic cancer cells [56], glioma cells [57, 58]. In the present study, we demonstrated that inhibition or silence of BK_Ca_ decreased, while inhibition of I_Kir_ enhanced migrating ability of human cardiac c-kit^+^ progenitor cells, indicating that BK_Ca_ promotes, while I_Kir_ inhibits, the cell migration in human cardiac c-kit^+^ progenitor cells under physiological conditions. It should be noted that the observed effects of the BK_Ca_ channel or Kir2.1 channel blocker tested here may be related to the experimental condition used in this particular set of experiments (for instance the incubation time), however, the results from experiments with specific siRNA molecules indicate the more specific effects in this specific cell type.

Collectively, the present study provided the novel information that under physiological conditions BK_Ca_, but not I_Kir_, may promote cell proliferation and cell mobility, while I_Kir_ could inhibit cell migration without affecting proliferation. I_Na.TTX_ has no effect on cell proliferation or migration. The information provides a base for the further understanding of cellular physiology and biology in human cardiac c-kit^+^ progenitor cells.

## Supporting Information

S1 File Ethic approval letter. (PDF)
