# Supplementary material for: Effects of BKCa and Kir2.1 Channels on Cell Cycling Progression and Migration in Human Cardiac c-kit+ Progenitor Cells
Source: PLoS One. 2015 Sep 21;10(9):e0138581. doi: 10.1371/journal.pone.0138581 (PMC4577111; doi:10.1371/journal.pone.0138581)
Supplement: S1 File — (PDF) [file pone.0138581.s001.pdf]

香港大學
University of Hong Kong
香港大學及醫管局港島西醫院聯網研究倫理委員會
醫院管理局
HOSPITAL AUTHORITY

Institutional Review Board of the University of Hong Kong/ Hospital Authority Hong Kong West Cluster (HKU/HA HKW IRB)

Address: Rm 901, Administration Block, QMH Tel 2255 3923 2255 4086 Fax 2255 4735

Dr. Gui-Rong Li
Medicine
(L4-59 Lab Block, FMB), HKU
20-Apr-10

Dear Dr. Li,

IRB Reference Number: **UW 10-174**

The HKU/HA HKW IRB is authorized by a joint agreement of the University of Hong Kong and Hospital Authority Hong Kong West Cluster to review and monitor clinical research. It serves to ensure that research complies with the Declaration of Helsinki and acts in accordance to ICH GCP guidelines, local regulations and Hospital Authority and the University policies.

I write to inform that your research application/submission has been approved by an expedited process with details shown below. You are also requested to adhere to the conditions listed.

**Protocol title**	:	Collection of Human Cardiac Atrial Specimens for Cellular Electrophysiological Study
**Study site(s)**	:	As stated in application form
**IRB reviewer**	:	Professor Virginia Wong, Chairman of the HKU/HA HKW IRB
**Document(s) approved**	:	01. Clinical research ethics review application form
	:	02. Research protocol 1.0
	:	03. Research information for patient - English and Chinese
	:	04. Patient / Subject consent form - English and Chinese
**Document(s) reviewed**	:	05. Short CV of principal investigator

(Conditions :
1. Do not deviate from, or make changes to the study protocol without prior written IRB approval, except when it is necessary to eliminate immediate hazards to research subjects or when the change involves only logistical or administrative issues.
2. Report the following to HKU/HA HKW IRB: (i) study protocol or consent document change (use 'HKU/HA HKW IRB RE001F7'), (ii) serious adverse event (use 'HKU/HA HKW IRB RE001F8'). (iii) study progress (use 'HKU/HA HKW IRB RE001F9a')* (iv) new information that may be relevant to a subject's willingness to continue participation in the study.
3. Report study progress to HKU/HA HKW IRB at a 12-monthly interval until study closure.)

Yours sincerely,

W. H. Lee
HKU/HA HKW IRB Secretary
